# Morphological Investigation of the Roe Deer (*Capreolus capreolus*) Os Hyoideum and Creating a 3D Model

**DOI:** 10.1002/vms3.70267

**Published:** 2025-05-23

**Authors:** Sedef Selviler Sizer, Fatmanur Sıla Keskin, Semih Kurt, Betül Kanik, Yonca Betil Kabak, Burcu Onuk, Murat Kabak

**Affiliations:** ^1^ Department of Anatomy, Faculty of Veterinary Medicine Ondokuz Mayis University Samsun Turkey; ^2^ Department of Veterinary Anatomy, Graduate Education Institute Ondokuz Mayis University Samsun Turkey; ^3^ Department of Pathology, Faculty of Veterinary Medicine Ondokuz Mayis University Samsun Turkey

**Keywords:** 3D printing, anatomy, bone, osteometry, roe deer

## Abstract

The os hyoideum, located at the intersection of the respiratory and digestive tracts, holds strategic importance due to its role in breathing, swallowing and vocalisation. The morphological structure of the os hyoideum varies among species. Since no research has been found on the hyoid bone in the roe deer (*Capreolus capreolus*), this study was planned. Six roe deer os hyoideum were used. The bones forming the os hyoideum were examined macroscopically and histologically, with morphometric measurements taken from 28 parameters. Additionally, the roe deer os hyoideum was 3D modelled and printed using TPU filament. The hyoid bone, connected to the skull by syndesmosis, consists of six parts: basihyoideum, thyrohyoideum, ceratohyoideum, epihyoideum, stylohyoideum and tympanohyoideum. While the tympanohyoideum is entirely compact bone, the middle of the other parts is compact bone and the ends are spongy bone. Among the joints between the parts, the tympanohyoideum—stylohyoideum and basihyoideum—thyrohyoideum joints are synchondrotic, while the others are synovial. In the roe deer, stylohyoideum was the longest part at 42.56 mm, while tympanohyoideum was the smallest at 6.92 mm. When the os hyoideum was printed using TPU filament, each bone part maintained its structural integrity. This study provided detailed morphological and morphometric data on the roe deer os hyoideum for the first time. The absence of proc. lingualis in the basihyoideum and the general morphological resemblance to carnivora in roe deer were noteworthy.

## Graphical abstract

With this study, detailed morphological, morphometric and 3D printing data of the roe deer os hyoideum were obtained for the first time and its similarities and differences with other animal species were compared. The os hyoideum consisted of six parts, but the processus lingualis, which is found in ruminants, was not present in the roe deer. When the general morphological structures and morphometric values of the os hyoideum were examined, their similarities with carnivorous species were striking.

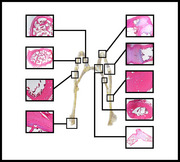

## 1 Introduction

Most of the bones that make up the cranium are flat bones with intramembranous ossification, while the base of the cranium consists of irregular bones with intracartilaginous ossification (König and Bragulla 2007). Many of these bones are connected to each other by sutures or synchondrosis joints (Nejdet 2007). Among these, the mandible forms a diarthrosis joint with the os temporale, while the os hyoideum forms either a synchondrosis or syndesmosis joint with the same bone, varying depending on the animal species (Bahadir and Yildiz 2016; Sisson et al. 1975). The os hyoideum is located between the two rami of the mandible and extends caudally from the dorsal portion (Sisson et al. 1975). This bone is formed by the articulation of small bones consisting of flat, round fibrocartilaginous and osseous tissues (Nejdet 2007). The bones constituting the os hyoideum are divided into two sections. The first section, known as the lingual apparatus, includes the basihyoideum, thyrohyoideum and ceratohyoideum, which are connected to the tongue and larynx. The second section, functioning as the suspensory apparatus of the tongue, consists of the epihyoideum, stylohyoideum and tympanohyoideum (Bahadir and Yildiz 2016; Nejdet 2007; Sisson et al. 1975). The structure, shape, size and methods of connection of the bones that make up the os hyoideum to each other and the skull vary among animal species (Bahadir and Yildiz 2016; Hilloowala 1975; König and Bragulla 2007; Nejdet 2007; Ross et al. 2024; Sisson et al. 1975; Weissengruber et al. 2002). The os hyoideum is an anatomical structure that, despite many studies on its function, still retains its mystery (Ross et al. 2024). This bone plays significant roles across a wide biomechanical and functional spectrum, ranging from feeding habits to sound production in animals (Bosma 1956; Ross et al. 2024; Smith 1992). The os hyoideum connects to the mandible, lingua, larynx, pharynx, sternum and cranium through muscles and ligaments (Shoshani and Marchant 2001). The attachment of the os hyoideum to the cranium via muscles and ligaments facilitates the control of laryngeal position, creating a direct impact on vital functions such as swallowing, breathing, sound production and feeding (Anapol 1988; Bosma 1956). Furthermore, in species where the os hyoideum forms a complete bony chain, it provides stabilisation for the tongue and pharyngeal muscles, playing a crucial role in the swallowing mechanism during feeding (Bosma 1956; Ross et al. 2024; Smith 1992). Directly examining the anatomical structure of the os hyoideum allows us to understand the physical characteristics of the bone (its shape, dimensions, surface features and articulation points), while 3D modelling techniques provide a digital representation to further explore the concrete structure, unique geometric form and relationships with surrounding structures (Bakıcı et al. 2019; Fakhry et al. 2013). These two approaches work in parallel and complement each other: data obtained through anatomical observations form the foundation for the 3D modelling process, while the visual and spatial depth offered by modelling aids in a more comprehensive understanding of the bone structure in the real world (Dayan and Besoluk 2011). The data used in the modelling process (e.g., CT scans or other imaging techniques) are compared with physical observations to verify whether the model accurately represents the actual anatomical structure (de Freitas et al. 2011; Hackmann et al. 2019; Li et al. 2018). By integrating both physical and digital data, the reliability of the model is enhanced (Li et al. 2018). Additionally, 3D models serve as precise tools for analysing the functional adaptations of the examined structures and, when combined with simulation techniques, contribute to illuminating evolutionary history (Porro et al. 2015). With advancing technology, anatomical structures with biomechanical properties, such as the os hyoideum, are being modelled digitally, making them more accessible and detailed (Bakıcı et al. 2019; Wilhite and Wölfel 2019). The 3D modelling of the os hyoideum not only facilitates a clearer understanding of its anatomical features but also enables more effective functional analyses and clinical interventions. Today, anatomical materials are designed using 3D devices and transformed into physical models through 3D printing technologies (Wilhite and Wölfel 2019). Different filaments such as Polylactic Acid (PLA), Thermoplastic Polyurethane (TPU) and Acrylonitrile Butadiene Styrene (ABS) are used with these printers (Altunkaynak et al. 2020; Şahin and Turan 2018). The suitability of these filaments is determined based on the requirements of the model to be printed, considering their advantages and disadvantages (Desai et al. 2023). While TPU provides a reliable option for more delicate and flexible structures, filaments like PLA exhibit more brittle characteristics (Bakıcı et al. 2019). The flexibility advantage offered by TPU filament ensures the long‐term safe use of fragile and hard‐to‐access anatomical materials (Kurt et al. 2022; Şahin and Turan 2018).

In the literature, studies on the morphology of the os hyoideum have been conducted on various animal species such as giant anteater (Borges et al. 2017), domestic cat (Farag and El‐Saba 2013), green turtle (Lopes et al. 2019), camel (El‐Shaieb and Majeed 1979; Shoghy and Saber 2013), guinea fowl, turkey (İlgün et al. 2015), awassi sheep (Ahmed and Mahmood 2013), hamster (Sprague 1941), toothed whale (Reidenberg and Laitman 1994), bison, elk, moose, caribou, domestic sheep, mountain goat and white‐tailed deer (Hale 2016). There are even studies in different fields related to the os hyoideum, such as its foetal development (Ahmed and Mahmood 2013; Shoghy and Saber 2013), histology (Farag and El‐Saba 2013), ontogenetic development (Weissengruber et al. 2002), comparison of terrestrial and aquatic mammals (Reidenberg and Laitman 1994), 3D modelling and PLA filament printing (Bakıcı et al. 2019). However, literature reviews have revealed no studies on the os hyoideum of the roe deer (*Capreolus capreolus*), a species belonging to the deer family (Cervidae) of the even‐toed ungulates order (Artiodactyla), which holds significant importance in wildlife (Sempéré et al. 1996). The aim of this study is to examine the morphological features of the os hyoideum of the roe deer species for the first time, to provide morphometric data and to model this structure in three dimensions (3D). The obtained models will allow examination in a digital environment and offer a solution to the problem of limited material access. At the same time, by making these 3D models usable as educational materials, durable and reproducible resources, it aims to address the deficiency in the related literature and to establish an important basis for future biomechanical and clinical studies.

## 2 Material and Methods

**FIGURE 1:**
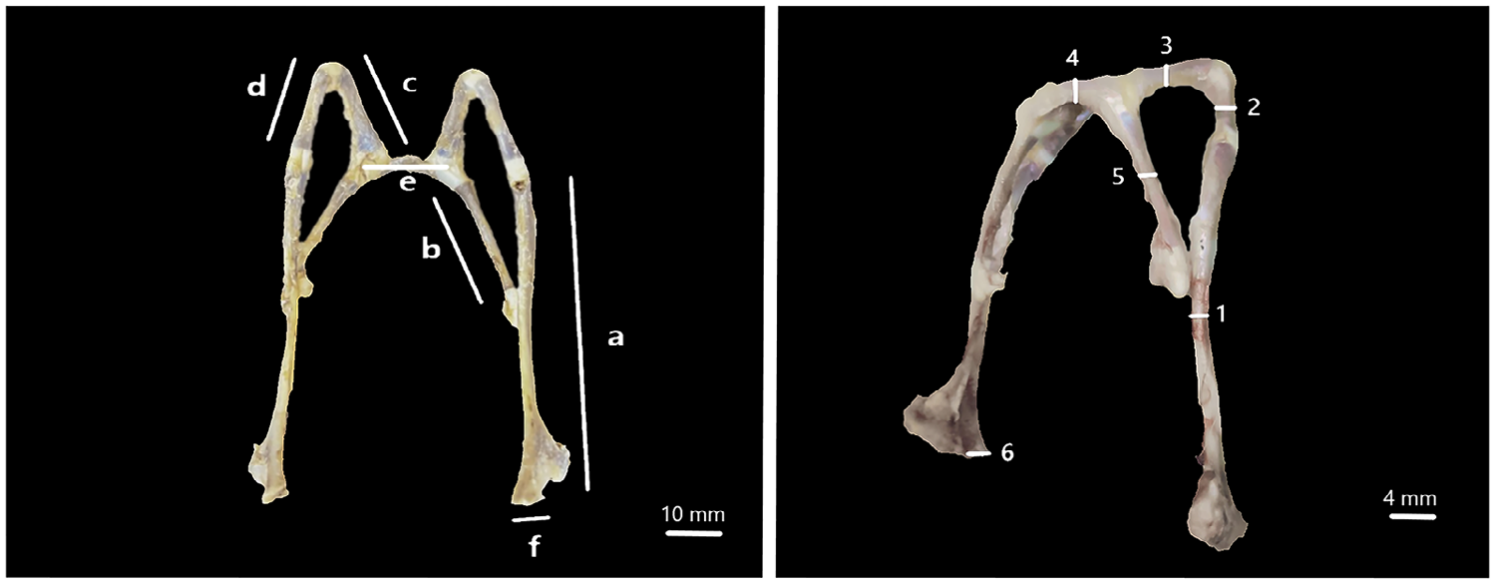
Measurement parameters for the length and thickness of the os hyoideum. (a) Length of stylohyoideum, (b) length of thyrohyoideum, (c) length of ceratohyoideum, (d) length of epihyoideum, (e) length of basihyoideum, (f) length of tympanohyoideum, (1) thickness of stylohyoideum, (2) thickness of epihyoideum, (3) thickness of ceratohyoideum, (4) thickness of basihyoideum, (5) thickness of thyrohyoideum and (6) thickness of tympanohyoideum.

**FIGURE 2:**
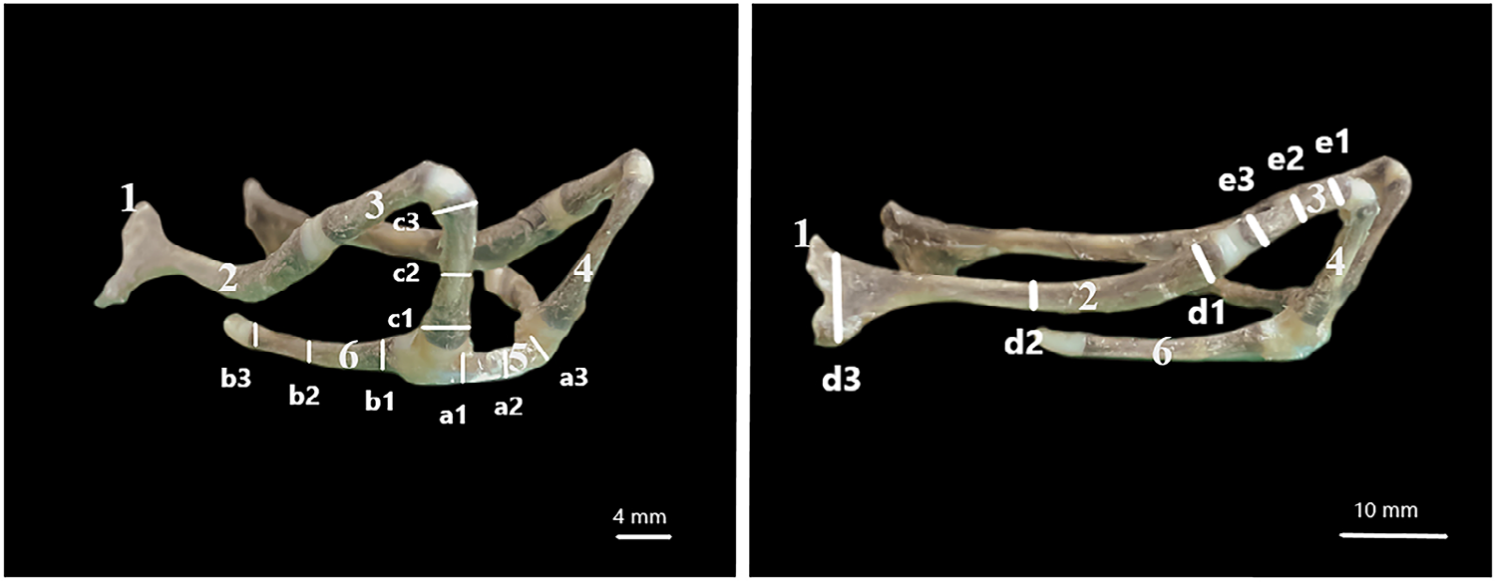
The tip height, middle height and end height measurement points of os hyoideum parts. (1) tympanohyoideum, (2) stylohyoideum, (3) epihyoideum, (4) ceratohyoideum, (5) basihyoideum, (6) thyrohyoideum. (a1) tip height of basihyoideum, (a2) middle height of basihyoideum, (a3) end height of basihyoideum, (b1) tip height of thyrohyoideum, (b2) middle height of thyrohyoideum, (b3) end height of thyrohyoideum, (c1) tip height of epihyoideum, (c2) middle height of epihyoideum, (c3) end height of epihyoideum, (d1) tip height of stylohyoideum, (d2) middle height of stylohyoideum, (d3) end height of stylohoideum, (e1) tip height of ceratohyoideum, (e2) middle height of ceratohyoideum and (e3) end height of ceratohyoideum.

In this study, the hyoideums of six adult roe deer (*Capreolus capreolus*) that could not be saved for various reasons and whose gender was disregarded and were brought to the faculty clinics were dissected from cadavers. Four of the materials were cleaned in approximately 10 h using Dermestes maculatus larvae. The remaining two materials were dissected to be used in histological examinations, fixed in 10% formaldehyde solution for 24 h and then followed in decalcification solution. All the materials were first examined macroanatomically. Taking into account the studies conducted by Farag and El‐Saba (2013), Selim et al. (1984), Weissengruber et al. (2002) and Hale (2016), a total of 28 parameters were measured, including length, thickness (Figure 1) and height (Figure 2) in the basihyoideum, thyrohyoideum, ceratohyoideum, epihyoideum, stylohyoideum and tympanohyoideum regions. Arithmetic means and standard deviations of the measurements for length, thickness, tip height, middle height and end height were calculated. Morphometric data were obtained using a digital calliper (Mitutoyo, Japan). The os hyoideum was photographed with an iPhone. Nomina Anatomica Veterinaria (Nomenclature 2017) was used for nomenclature.

The two materials fixed for histological examination were placed in a solution consisting of 20% sodium citrate (w/v) and 45% formic acid (v/v) for decalcification (Morse 1945). The decalcification process was followed by changing the solution daily until the bones were decalcified. After this process, which lasted approximately two weeks, the bones were kept in bicarbonate buffer solution for 6 h. The decalcified materials were embedded in paraffin using routine tissue procedures. Serial sections of 5 µm thickness were taken from the embedded samples using a Leica (RM2125RT) microtome and stained with Haematoxylin‐Eosin (H&E) (Luna 1968). Histological examination was performed using a Nikon Eclipse E600 W light microscope and microscopic photographs were taken with the Nikon DS camera head DS‐5 M imaging system.

**FIGURE 3:**
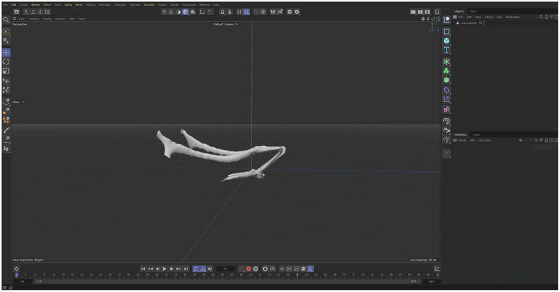
Polygon arrangement roe deer os hyoideum in cinema 4D after 3D scanning.

For 3D examination, the os hyoideum of the roe deer was scanned using a 3D scanner (creality lizard). 3D models were created from the images and saved in stl format. The model was transferred to Cinema 4D for polygon editing (Figure 3). The created 3D models were printed using a fused deposition modelling (FDM) type 3D printer (creality nder 5‐S1) with TPU filament to produce a flexible material (Table 1).

**TABLE 1:** Printing parameters targeted for 3D printing of TPU filaments.

Print Settings	TPU
Nozzle diamater	**0.4mm**
Press temperature	**230°C**
Bed temperature	**70**°**C**
Fan speed	**100%**
Internal occupancy	**20%**
Print speed	**50mm/sn**
Retraction	**—**
Layer thickness	**0.12mm**

*Note*: Printing speed, nozzle and table temperature values vary depending on filament and 3D printer brands.

## 3 Results

**FIGURE 4:**
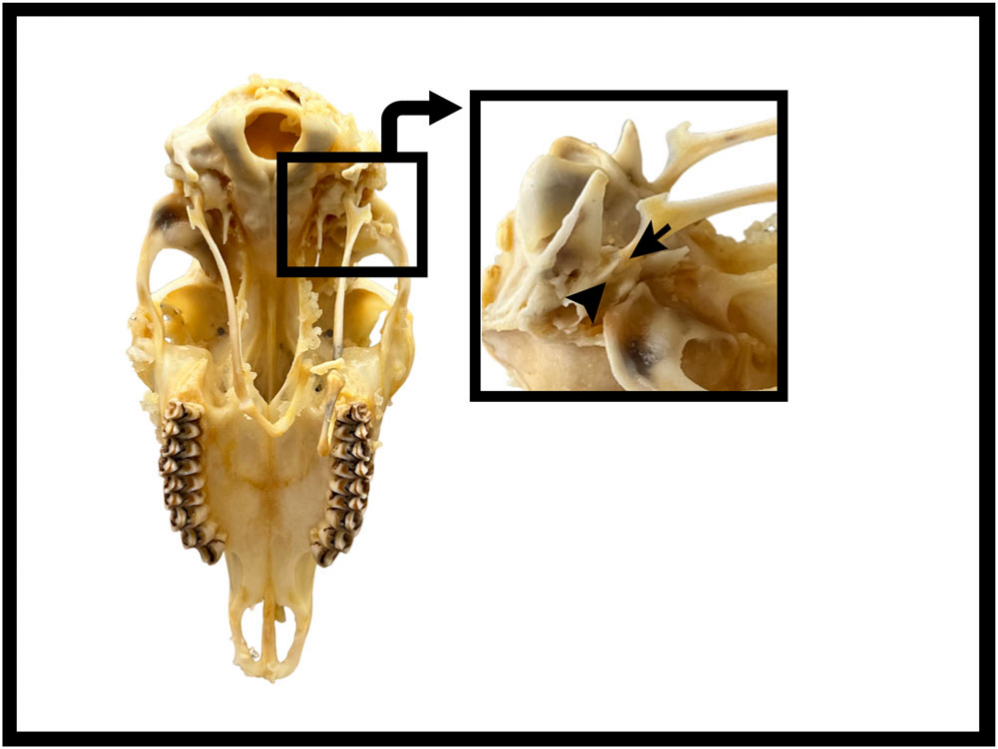
Connection of the hyoid bone of the roe deer to the base of the cranium. Tympanohyoideum (black arrow) processus styloideus of the os temporale (arrowhead).

**FIGURE 5:**
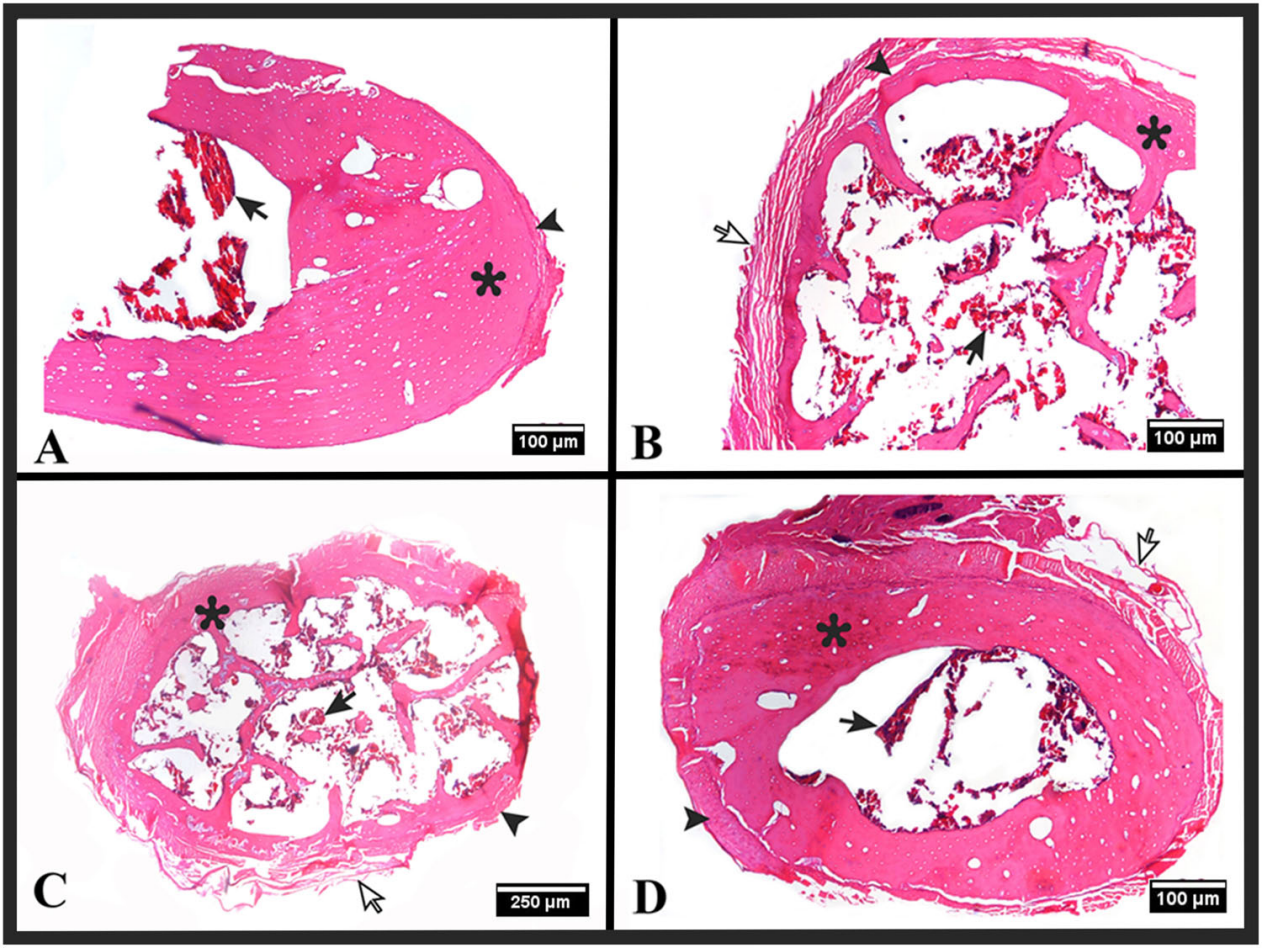
Histology of tissue sections taken from the midline of bone fragments of the os hyoideum. H and E stain. (A) Stylohyoideum (4x objective magnification), (B) ceratohyoideum (4x objective magnification), (C) epihyoideum (2x objective magnification) and (D) thyrohyoideum (4x objective magnification). Compact bone tissue (*), bone periosteum (arrowhead), connective tissue (white arrow) and bone marrow (black arrow).

**FIGURE 6:**
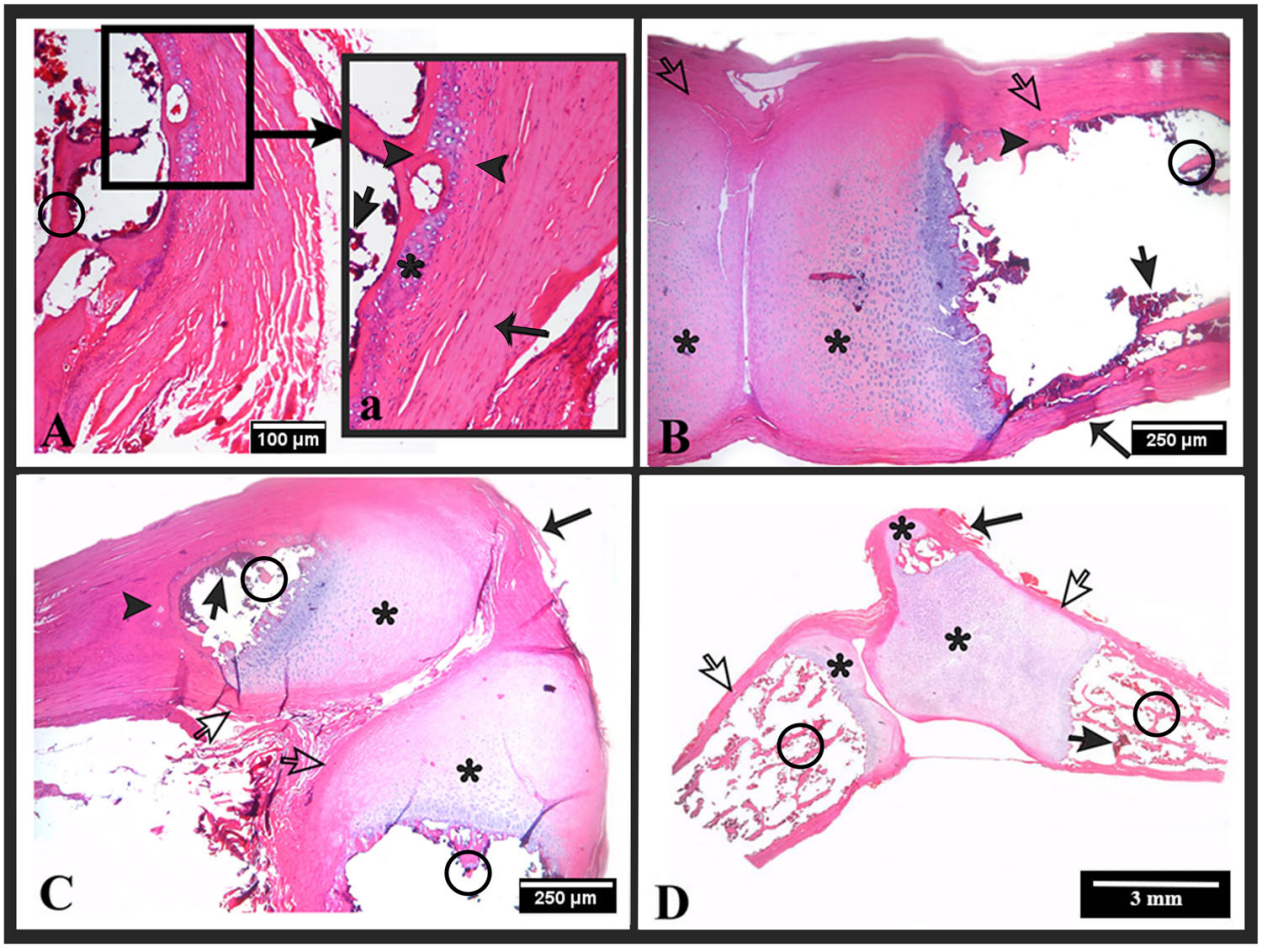
Histology of tissue sections taken from the joints of the os hyoideum. H and E stain. (A) Tympanohyoideum—stylohyoideum joint (4x objective magnification), (a) tympanohyoideum—stylohyoideum joint (10x objective magnification), (B) stylohyoideum—epihyoideum joint (2x objective magnification), (C) epihyoideum—ceratohyoideum joint (2x objective magnification), (D) ceratohyoideum—basihyoideum and basihyoideum—thyrohyoideum joints (1x objective magnification). (a) and (c) stylohyoideum, (b) tympanohyoideum, (d) and (e) epihyoideum, (f) and (g) ceratohyoideum, (h) basihyoideum and (i) thryohyoideum. Hyaline cartilage (*), compact bone tissue (arrowhead), spongy bone tissue (circle), bone periosteum (white arrow), bone marrow (black arrow) and connective tissue (thin arrow).

In the roe deer, the basal part of the os hyoideum consists of the basihyoideum and thyrohyoideum, while the suspensory part consists of the tympanohyoideum, stylohyoideum, ceratohyoideum and epihyoideum (Figures 1 and 2). The basihyoideum is positioned transversely at the level of the cranium base and has a slightly convex curve in shape. There was no processus lingualis observed on this part. The ceratohyoideum, which articulates with the basihyoideum and extends dorsally, is a short cylindrical bone. It continues at a narrow angle to articulate with the epihyoideum. The epihyoideum, extending caudal, is nearly identical to the ceratohyoideum in both shape and length. The stylohyoideum, located between the epihyoideum and tympanohyoideum, is observed as a flat rod like bone that slightly widens caudally. The tympanohyoideum is a very small projection continuing from the stylohyoideum. This bone forms a syndesmosis joint with the processus styloideus of the os temporale in the cranium (Figures 4, 5, 6).

The measurements of the bones that constitute the os hyoideum are shown in Tables 2, 3, 4, 5, 6. According to the data in Table 2, the average lengths of the basihyoideum, ceratohyoideum and epihyoideum were found to be similar. When evaluating the length data of all parts, it was revealed that the stylohyoideum is the longest part while the tympanohyoideum is the shortest. Regarding the arithmetic mean and standard deviation values of the bone thicknesses (Table 3), it was determined that the thicknesses of the thyrohyoideum and stylohyoideum are similar. The basihyoideum was the thickest bone, whereas the stylohyoideum was the thinnest. Height measurements for each bone were obtained from three different points: tip, middle and end (Figure 2). Considering the tip height data (Table 4), the values of the basihyoideum and thyrohyoideum were similar, as were the values of the ceratohyoideum and epihyoideum. The stylohyoideum had the highest tip height, while the thyrohyoideum had the lowest. According to the middle height data (Table 5), the height of the thyrohyoideum was similar to that of the epihyoideum. Additionally, the stylohyoideum was found to have the highest middle height, while the thyrohyoideum had the lowest. Examining the end height data (Table 6), the arithmetic mean value for the stylohyoideum was significantly higher than for the other parts. The thyrohyoideum had the lowest end height average. Based on these results, considering the tip height, middle height and end height data, it was observed that the stylohyoideum was the highest part for all three parameters, while the thyrohyoideum was the lowest part. The arithmetic means of the tip, middle and end heights of each part of the os hyoideum in Table 7 also supported this finding. These data indicated that the stylohyoideum was the highest part along the bone length, while the thyrohyoideum was the lowest.

**TABLE 2:** Arithmetic mean and standard deviation data of os hyoideum length measurements.

	Basihyoideum	Thyrohyoideum	Ceratohyoideum	Epihyoideum	Stylohyoideum	Tympanohyoideum
Length (X̅ ± SD mm)	14,03 ± 1,95	29,76 ± 1,58	17,61 ± 2,45	15,22 ± 1,37	42,56 ± 4,55	6,92 ± 0,86

Abbreviations: SD, Standard deviation; X̅, Arithmetic mean.

**TABLE 3:** Arithmetic mean and standard deviation data of os hyoideum thickness.

	Basihyoideum	Thyrohyoideum	Ceratohyoideum	Epihyoideum	Stylohyoideum	Tympanohyoideum
Thickness (X̅ ± SD mm)	3,71 ± 0,79	1,48 ± 0,15	1,89 ± 0,45	1,69 ± 0,26	1,45 ± 0,41	2,72 ± 0,32

Abbreviations: SD, Standard deviation; X̅, Arithmetic mean.

**TABLE 4:** Arithmetic mean and standard deviation data of os hyoideum tip height measurements.

	Basihyoideum	Thyrohyoideum	Ceratohyoideum	Epihyoideum	Stylohyoideum	Tympanohyoideum
Tip height (X̅ ± SD mm)	3,18 ± 0,51	3,13 ± 0,53	3,37 ± 0,69	3,38 ± 0,75	4,02 ± 0,31	3,47 ± 0,47

Abbreviations: SD, Standard deviation; X̅, Arithmetic mean.

**TABLE 5:** Arithmetic mean and standard deviation data of os hyoideum middle height measurements.

	Basihyoideum	Thyrohyoideum	Ceratohyoideum	Epihyoideum	Stylohyoideum	Tympanohyoideum
Middle height (X̅ ± SD mm)	2,53 ± 0,61	2,33 ± 0,38	2,43 ± 0,28	2,36 ± 0,38	3,10 ± 0,36	*

Abbreviations: SD, Standard deviation; X̅, Arithmetic mean.

* Measurement without data.

**TABLE 6:** Arithmetic mean and standard deviation data of os hyoideum end height.

	Basihyoideum	Thyrohyoideum	Ceratohyoideum	Epihyoideum	Stylohyoideum	Tympanohyoideum
End height (X̅ ± SD mm)	3,52 ± 0,72	2,53 ± 0,29	4,12 ± 0,22	3,54 ± 0,28	9,64 ± 1,41	*

Abbreviations: SD, Standard deviation; X̅, Arithmetic mean.

* Measurement without data.

**TABLE 7:** Arithmetic mean and standard deviation values of each bone of the os hyoideum along its length (calculated based on tip, middle and end height values).

	Basihyoideum	Thyrohyoideum	Ceratohyoideum	Epihyoideum	Stylohyoideum	Tympanohyoideum
X̅ ± SD mm	3,07 ± 0,50	2,66 ± 0,41	3,30 ± 0,84	3,09 ± 0,64	5,58 ± 3,54	^*^

* Measurement without data.

Histological examinations revealed that the tympanohyoideum is entirely composed of compact bone tissue. Sections taken from the midline of the stylohyoideum, epihyoideum, ceratohyoideum, basihyoideum and thyrohyoideum showed compact bone tissue (Figure 5), while sections taken from the ends of these bones showed spongy bone tissue (Figure 6). Two different types of joints, synchondrosis and synovial, were identified between the bones. The synchondrotic joints were found between the tympanohyoideum and stylohyoideum, and between the basihyoideum and thyrohyoideum. The synovial joints were found between the stylohyoideum and epihyoideum, epihyoideum and ceratohyoideum, and ceratohyoideum and basihyoideum (Figure 6).

As a result of the roe deer os hyoideum model being produced using TPU filament using the 3D printing method, it was determined that it exactly reflects the anatomical features of the real os hyoideum. Thanks to the flexible structure of the TPU filament, it was determined that the model could return to its original form after stretching during use. In addition, it was observed that the filament could be used safely for thin and delicate anatomical structures.

## 4 Discussion

The number of parts constituting the bone structure of the os hyoideum varies among animal species. In rabbits (Farag et al. 2012), this bone consists of three parts: basihyoideum, thyrohyoideum and ceratohyoideum. The presence of the tympanohyoideum is not mentioned in giant anteaters (Borges et al. 2017). In many species, including domestic mammals (König and Bragulla 2007; Sisson et al. 1975), camel (El‐Shaieb and Majeed 1979; Shoghy and Saber 2013), lion, jaguar, tiger, cheetah, domestic cat (Weissengruber et al. 2002) and toothed whale (Reidenberg and Laitman 1994), the os hyoideum has been reported to consist of six parts: basihyoideum, thyrohyoideum, ceratohyoideum, epihyoideum, stylohyoideum and tympanohyoideum. In the present study, the fact that the os hyoideum in roe deer consists of six parts is consistent with the literature.

The basihyoideum, which forms part of the os hyoideum, varies in shape and location among different animal species (Sisson et al. 1975). While the position of the basihyoideum has been reported to be at the level of the C3–C5 cervical vertebrae in lions, tigers and adult jaguars (Weissengruber et al. 2002), in the roe deer in our study, it was observed to be located ventral to the base of the skull or the atlas, similar to that in cheetahs and domestic cats. In the camel (Shoghy and Saber 2013), cat and dog (Sisson et al. 1975), this structure has been reported to appear as a slightly curved crossbar without a processus lingualis. In sheep, a short and straight processus lingualis has been reported, while in oxen, a short and tubular processus lingualis has been reported, centrally located on the rostral side of the basihyoideum (Sisson et al. 1975). In pigs, the basihyoideum has been reported to be rostrocaudally broad, transversely very short and bearing a pointed processus lingualis (Shands Jr 1955; Sisson et al. 1975). In the horse (Sisson et al. 1975), the basihyoideum is a short crossbar with a long processus lingualis, whereas in the rabbit (Farag et al. 2012), the basihyoideum is wedge‐shaped with a very short projection known as the processus lingualis. In buffalo, the basihyoideum is defined as a short transverse rod with a thick rostral side and a thin caudal side, containing a tubercle‐shaped processus lingualis (Selim et al. 1984). In our study, it was found that the basihyoideum of the roe deer, a wild ruminant, was positioned transversely with a slightly convex curve and no processus lingualis was found. Nazih (2019) reported in his research to define the relationship between the os hyoideum anatomy of different species and feeding behaviours that in cattle and buffalo, the m. hyoglossus, m. geniohyoideus and m. genioglossus muscles start from the proc. lingualis, in camels, due to the absence of proc. lingualis, m. hyoglossus starts from the basihyoideum and thyrohyoideum, m. geniohyoideus starts from the thyrohyoideum and m. genioglossus has no connection with the hyoid bone. He also associated the stronger tongue structure of cattle and buffalo compared to camels with this anatomical difference. Based on this literature information, the study suggested that the absence of proc. lingualis in roe deer, unlike species that actively use their tongues during grass‐gathering behaviour, may be an evolutionary adaptation due to the more active use of their lips and teeth.

When examining the morphometric data of the parts of the os hyoideum, it has been reported that the longest part in the lion is the epihyoideum, while in the tiger it is the thyrohyoideum (Weissengruber et al. 2002). In our study, the stylohyoideum of the roe deer was determined to be the longest part among all the components. This finding is similar to that observed in the giant anteater (Borges et al. 2017), toothed whale (Reidenberg and Laitman 1994), jaguar, cheetah, domestic cat (Weissengruber et al. 2002) and buffalo (Selim et al. 1984). When comparing the lengths of the stylohyoideum, it was found that the stylohyoideum of the roe deer (46.52 mm) is longer than that of the lion (28 mm), tiger (36 mm), cheetah (32 mm) and domestic cat (12 mm) (Weissengruber et al. 2002), but shorter than that of the domestic sheep (59.83 mm), bighorn sheep (65.05 mm), dall sheep (64.83 mm), domestic goat (58 mm), mountain goat (81.95 mm), mule deer (71.35 mm), white‐tailed deer (74.29 mm), pronghorn antelope (77.60 mm) (Hale 2016) and buffalo (120–130 mm) (Selim et al. 1984). Notably, the lengths of the stylohyoideum in roe deer were similar to that of the jaguar (41–44 mm) (Weissengruber et al. 2002).

In the os hyoideum, the shortest part has been reported to be the ceratohyoideum in the giant anteater (Borges et al. 2017), lion (16 mm), tiger (13 mm), jaguar (F: 12 mm, M: 16 mm) and domestic cat (5 mm), while in the cheetah (16 mm), the shortest bone part is described as the tympanohyoideum (Weissengruber et al. 2002). El‐Shaieb and Majeed (1979) reported that the shortest part in the camel is the basihyoideum. In the buffalo, the shortest parts of the os hyoideum are reported to be the epihyoideum (15– 20 mm) and the basihyoideum (15– 20 mm) (Selim et al. 1984). In this study, the shortest part in the roe deer was found to be the tympanohyoideum, measuring 6.92 mm, which is similar to the finding reported in the cheetah (Weissengruber et al. 2002).

As a result of the studies, when the parts that make up the os hyoideum were compared with each other, the epihyoideum was found to be the most significantly different in size among the species. It has been reported that in the giant anteater (Borges et al. 2017), the epihyoideum is about half the length of the stylohyoideum. In the lion and tiger (Weissengruber et al. 2002), the length of the epihyoideum is approximately 3–4 times that of the ceratohyoideum, while in the camel (El‐Shaieb and Majeed 1979) and domestic cat (Farag and El‐Saba 2013), it is reported to be twice as long. In the buffalo, the ceratohyoideum is almost twice as long as the epihyoideum (Selim et al. 1984). Additionally, it was observed that in the domestic cat (Farag and El‐Saba 2013), the length of the epihyoideum is quite similar to the lengths of the stylohyoideum, tympanohyoideum, basihyoideum and thyrohyoideum. In the horse, the epihyoideum is found in small pieces, while in cattle, it is nearly as long as the ceratohyoideum (Sisson et al. 1975). In the roe deer, similar to the cheetah (Weissengruber et al. 2002), the epihyoideum was determined to be approximately the same length as the ceratohyoideum and basihyoideum.

In the literature, it has been mentioned that in the domestic cat and lion, the tympanohyoideum is almost the same length as the stylohyoideum, while in the tiger, adult jaguar and cheetah, the tympanohyoideum is half the length of the stylohyoideum (Weissengruber et al. 2002). In our study, it was noteworthy that the tympanohyoideum (6.92 mm) in the roe deer was almost six times smaller than the stylohyoideum (42.56 mm).

The thickness values of the stylohyoideum have been measured in various species: cattle (5.66 mm), bison (4.39 mm), elk (3.62 mm), moose (3.54 mm), caribou (2.91 mm), horse (2.82 mm), domestic sheep (2.19 mm), mountain goat (2.84 mm), white‐tailed deer (2.11 mm), bighorn sheep (1.80 mm), dall sheep (1.87 mm), domestic goat (1.80 mm), mule deer (1.85 mm), pronghorn antelope (1.72 mm) (Hale 2016), tiger (3.5 mm), cheetah (4– 5 mm), lion (1– 2 mm), jaguar (1– 2mm) and domestic cat (0.9– 1 mm) (Weissengruber et al. 2002). In our study, the thickness of the roe deer stylohyoideum was determined to be 1.45 ± 0.41 mm. This data indicates that the roe deer stylohyoideum is thinner than those of cattle, bison, elk, moose, caribou, horse, domestic sheep, mountain goat, white‐tailed deer (Hale 2016), tiger and cheetah (Weissengruber et al. 2002), but similar to those of bighorn sheep, dall sheep, domestic goat, mule deer, pronghorn antelope (Hale 2016), lion, jaguar and domestic cat (Weissengruber et al. 2002).

There are various studies on the biomechanical role of the attachment types of the os hyoideum to the cranium base during feeding in different animal species (Anapol 1988; Olson et al. 2021; Olson, Montuelle, Curtis, et al. 2021; Ross et al. 2024). In his study on humans, monkeys and rabbits, (Anapol 1988) reported that the muscular attachment of the hyoid bone to the skull base facilitates control of the larynx position and that this flexibility affects vital functions such as swallowing, respiration, sound production and feeding. In domestic mammals known to have articulations to the skull base, it is mentioned that the hyoid bone supports the stabilisation of the tongue and pharynx muscles and that this situation plays an important role in the swallowing mechanism during feeding (Bosma 1956; Ross et al. 2024; Smith 1992). The attachment site and shape of the os hyoideum to the base of the cranium vary among animal species (Anapol 1988; Bosma 1956; König and Bragulla 2007; Nejdet 2007). In equidae, ruminant (Bahadir and Yildiz 2016), camel (El‐Shaieb and Majeed 1979), lion, cheetah, tiger, jaguar (Weissengruber et al. 2002) and buffalo (Selim et al. 1984), the os hyoideum attaches to the processus stylohyoideus of the pars tympanica of the os temporale via the tympanohyoideum. In carnivores, it connects to the processus mastoideus of the same bone, in pigs to the processus nuchalis of the squama temporalis (Bahadir and Yildiz 2016) and in the giant anteater (Borges et al. 2017), the stylohyoideum of the os hyoideum connects to the protuberantia occipitalis. In rabbits, the attachment type of the os hyoideum remains controversial; some researchers state that the ceratohyoideum articulates with the processus jugularis, while others suggest that it is connected solely through muscles (Anapol 1988; Farag et al. 2012). In the literature, the method of attachment of the os hyoideum to the cranium has been reported as synchondrosis in equidae, ruminant and pig (Bahadir and Yildiz 2016) and in camels (Hillmann 1975); syndesmosis in carnivores (Bahadir and Yildiz 2016); synsarcosis in the giant anteater (Naples 1999) and rabbit (Farag et al. 2012); and both syndesmosis and synsarcosis in lion, cheetah, tiger and jaguar (Weissengruber et al. 2002). In the present study, the attachment of the os hyoideum to the processus styloideus in roe deer was found to be similar to that reported in equidae, ruminant (Sisson et al. 1975), camel (Shoghy and Saber 2013), lion, cheetah, tiger, jaguar (Weissengruber et al. 2002) and buffalo (Selim et al. 1984). However, the attachment type being syndesmosis was similar to that of carnivoesr. This situation, which is similar for roe deer and carnivor, is thought to be a biomechanical adaptation to accelerate feeding processes in processes where food intake must be done quickly in nature.

It has been reported in the literature that the components forming the os hyoideum can vary in structure among different species or families within the same animal, including bone, cartilage, or connective tissue (Fürbringer and Braus 1922; Lykaki and Papadopoulos 1988; Nakano et al. 1988). Shoghy and Saber (2013) demonstrated in adult camel that only the basihyoideum part of the os hyoideum consists of cartilage, while the other parts are composed of bone tissue. In domestic cat, the tympanohyoideum and ceratohyoideum are mostly hyaline cartilage, the rostral part of the epihyoideum is hyaline cartilage and the caudal part is fibrous cartilage, the ends of the stylohyoideum are hyaline cartilage with the middle part being spongy bone tissue, the thyrohyoideum consists partially of hyaline cartilage (near the cartilago thyroidea) and partially of spongy bone tissue (near the basihyoideum) and the basihyoideum is entirely spongy bone tissue (Farag and El‐Saba 2013). It has been noted that in one year old lion and cheetah, the tympanohyoideum is composed of cartilage, whereas in tiger and adult jaguar, this part is calcified (Weissengruber et al. 2002). Pocock (1916) referred to the epihyoideum of lion, tiger and jaguar as having an elastic and collagenous ligamentous structure, naming this part ligamentum epihyoideum. Weissengruber et al. (2002) also revealed a small embedded bone structure within this ligament in lion. Additionally, it has been reported in the literature that in lion, tiger and adult jaguar, the basihyoideum and ceratohyoideum consist of bone tissue, while the ventral part of the thyrohyoideum consists of bone tissue and the dorsal part consists of cartilage tissue (Weissengruber et al. 2002). Reidenberg and Laitman (1994) demonstrated in toothed whale that the ceratohyoideum, epihyoideum and tympanohyoideum consist of cartilage tissue, while the stylohyoideum, basihyoideum and thyrohyoideum consist of bone tissue. The study observed in roe deer that all parts are composed of bone tissue, with the tympanohyoideum being completely compact in structure, while the other parts were compact along the midline and spongy at the ends.

Studies have reported that the components forming the os hyoideum articulate with each other in different ways within the same animal and among species (Farag and El‐Saba 2013; Reidenberg and Laitman 1994; Weissengruber et al. 2002). In domestic cat (Farag and El‐Saba 2013), cheetah, lion, tiger and jaguar, the tympanohyoideum and stylohyoideum exhibit synchondrosis articulation (Weissengruber et al. 2002), while in toothed whale (Reidenberg and Laitman 1994), they show a synovial joint. The articulation between the stylohyoideum and epihyoideum is synchondrosis in domestic cat (Farag and El‐Saba 2013) and cheetah (Weissengruber et al. 2002), while in toothed whale (Reidenberg and Laitman 1994), it is synarthrosis and in lion, tiger and jaguar (Weissengruber et al. 2002), it involves a ligamentous connection. The epihyoideum and ceratohyoideum are synchondrosis in domestic cat (Farag and El‐Saba 2013), synovial in cheetah (Weissengruber et al. 2002) and toothed whale (Reidenberg and Laitman 1994) and ligamentous in lion, tiger and jaguar (Weissengruber et al. 2002). The articulation between the ceratohyoideum and basihyoideum is synovial in domestic cat, cheetah, lion, tiger, jaguar (Weissengruber et al. 2002) and toothed whale (Reidenberg and Laitman 1994). The joint between the basihyoideum and thyrohyoideum shows the most variation among the mentioned animals. In domestic cat, cheetah and lion, the articulation between basihyoideum and thyrohyoideum is reported as synovial, in tiger it is synchondrosis on the lateral side and synovial on the medial side, while in jaguar (Weissengruber et al. 2002) and toothed whale (Reidenberg and Laitman 1994), it is synostosis. Borges et al. (2017) reported that in giant anteater, all parts of the os hyoideum articulate via synovial joints based on CT images showing radiolucent inter articular spaces. In roe deer, two different types of articulations were identified: synchondrosis and synovial between parts. The synchondrosis articulation between basihyoideum and thyrohyoideum in roe deer was similar to that reported in tiger (Weissengruber et al. 2002). The articulation between epihyoideum and ceratohyoideum in roe deer was similar to that in cheetah (Weissengruber et al. 2002) and toothed whale (Reidenberg and Laitman 1994), while the articulation between ceratohyoideum and basihyoideum was synovial, similar to domestic cat, cheetah, lion, tiger, jaguar (Weissengruber et al. 2002) and toothed whale (Reidenberg and Laitman 1994).

Bakıcı et al. (2019) created a 3D model of the os hyoideum from CT images of horse, dog and cattle, which was then printed using PLA filament. However, the researchers noted that despite being more robust than real bones, these models could still damage some complex anatomical details on the os hyoideum. In a study by Kurt et al. (2022), the flexibility and durability of TPU filament were demonstrated using an FDM printer. The roe deer os hyoideum model created with this method was observed to be more resistant to breakage compared to real bones, as reported in the literature (Kurt et al. 2022). Additionally, it was found that each delicate bone part forming the os hyoideum maintained its structural integrity when printed using this method.

## 5 Conclusion

As a result, detailed morphological and morphometric data of the roe deer os hyoideum were obtained for the first time through this study. The absence of the processus lingualis in the basihyoideum of the roe deer, which is a ruminant species, distinguished it from other ruminant species and this difference suggested that the feeding performance of the species may be a result of evolutionary adaptation. In addition, the similarity of general morphological structures and morphometric data to carnivorous species is remarkable. Also, the fact that the os hyoideum model, which was produced for the first time with TPU filament, can be converted to the original formula, thanks to its flexible structure, showed that it can be a suitable material for complex, thin and regular anatomical structures such as tongue distribution.

## Author Contributions

All authors (Sedef Selviler Sizer, Fatmanur Sıla Keskin, Semih Kurt, Betül Kanik, Yonca Betil Kabak, Burcu Onuk and Murat Kabak) contributed equally to the conceptualisation, design, material preparation, data collection and analysis of the study. All authors read and approved the final version of the manuscript.

## Ethics Statement

Since no live animals were used in the study, there is no need for an ethics committee.

## Conflicts of Interest

The authors declare no conflicts of interest.

## Data Availability

The datasets generated during and/or analysed during the current study are available from the corresponding author on reasonable request.
